# Gossypetin targets the liver-brain axis to alleviate pre-existing liver fibrosis and hippocampal neuroinflammation in mice

**DOI:** 10.3389/fphar.2024.1385330

**Published:** 2024-05-27

**Authors:** Cenlu Xu, Haoran Tai, Yanan Chu, Ye Liu, Jiacheng He, Yiran Wang, Bingyin Su, Shurong Li

**Affiliations:** Development and Regeneration Key Lab of Sichuan Province, Department of Histology and Embryology, Department of Pathology, Chengdu Medical College, Chengdu, China

**Keywords:** liver fibrosis, p38 MAPK, gossypetin, hippocampus, neuroinflammation

## Abstract

Liver fibrosis occurs in response to chronic damage and inflammation to the liver. Leaving untreated, it can lead to decreased liver function and can eventually progress to cirrhosis, a more advanced and irreversible state of liver damage. Clinical investigations showed that chronic liver disease associated with neurological symptoms including anxiety, depression, and cognitive decline. However, few therapeutic options are available for treating liver and related brain pathologies simultaneously. In this study, we aim to find therapeutic candidates that target the liver-brain axis. Gossypetin, a flavonoid from sedum, shows promising capability in treating liver and brain pathologies in CCl_4_-induced mouse model. Short term of gossypetin administration is sufficient to ameliorate impaired liver function and pre-existing liver fibrosis, suppress MKK3/6-p38 MAPK and p53 activation, and abolish the activation of hepatic stellate cells and Kupffer cells. Although we observe no neuronal loss in the brain of mice with liver fibrosis, we do observe astrogliosis and microglial activation in certain brain regions, especially the hippocampus. Brief gossypetin administration also shows potential in alleviating neuroinflammation in these regions. These results suggest that gossypetin can target the liver-brain axis and be a promising candidate for treating chronic liver fibrosis patients with neurological symptoms.

## 1 Introduction

Chronic liver diseases will form fibrous septa and progress to liver fibrosis in the later stage (Tanwar et al., 2020). The mechanisms include hepatocyte degeneration and necrosis, changing gene expressions (Zhu et al., 2018), the release of harmful cytokines causing microenvironmental oxidative stress (Gan et al., 2022), combined with inflammatory responses, such as phagocytosis and secretion function of macrophages (Wang et al., 2023). These cause the hepatic stellate cells (HSCs) located in the Disse’s space, changing from a quiescent state to an active state. Activated HSCs have the function of myofibroblasts, which migrate to the injury site and secret extracellular matrix (ECM) to form the fibrous septum (Kisseleva and Brenner, 2021). Persistent liver fibrosis leads to further damage to liver structure and function, resulting in irreversible cirrhosis or even liver failure (Turning our focus to liver fibrosis, 2023). More evidence confirms that liver fibrosis can be inhibited, such as through anti-inflammatory, anti-oxidative stress, and changing the status of HSCs (Kisseleva et al., 2012).

Mitogen-activated protein kinases (MAPKs) are within the Serine/Threonine kinase family, including ERK, p38, JNK and ERK5 (Chang and Karin, 2001; Johnson and Lapadat, 2002). When cells are stimulated by inflammation, cytokines or other stresses, MAP kinase kinase kinase (MKKK), MAP kinase kinase (MKK) and MAPK are activated successively (Chang and Karin, 2001). In mice with non-alcoholic fatty liver disease induced by high-fat diet, macrophage p38α/β expression is upregulated, promoting secretion of cytokines and transformation to M1 type (Zhang et al., 2019); the upregulated expression of p38γ/δ in hepatocytes is involved in lipid metabolism and steatosis through the regulation of AMPK and mTOR signaling pathways (González-Terán et al., 2016). p38 MAPK can also activate downstream p53 through phosphorylation in Ser15, Ser20 and other sites (Wu, 2004). Activated p53 regulates a series of genes that lead to distinct cellular responses including cell cycle arrest and cell death (Choisy-Rossi and Yonish-Rouach, 1998; Hafner et al., 2019).

The interaction of liver and central nervous system, known as the liver-brain axis, have received extensive attention recently (Butterworth, 2013). In a cross-sectional study, depression and anxiety were common in patients with liver cirrhosis (Hernaez et al., 2022). Clinical studies showed that patients with hepatic fibrosis had worse cognitive function, and neuroimaging analysis confirmed that regional grey matter volumes were reduced in these patients, mainly in the hippocampus, thalamus, ventral striatum and cerebellum regions (Jiang et al., 2023). Animal experiments showed that bile duct ligation (BDL) caused hyperammonemia and activation of microglia and astrocytes, and impaired cognitive and motor function (Rodrigo et al., 2010). Hyperammonemia also led to neuroinflammation in the hippocampus and prefrontal cortex and hippocampal neuronal apoptosis in an acute CCl_4_ mice model (Khan et al., 2019). However, few therapeutic options are available for treating liver and related brain pathologies simultaneously.

Gossypetin (GTIN) is one of the hex hydroxylated flavonoids naturally existing in the sedum family. It was reported that GTIN was a strong scavenger of O_2_^−^•, •OH and DPPH• free radicals (Chen et al., 2013; Khan et al., 2013), and a potent inhibitor of MKK3/6-p38 MAPK signaling pathway (Xie et al., 2019). Previous studies showed that GTIN is hepatoprotective as it could ameliorates ionizing radiation-induced liver damage (Khan et al., 2015) and preventing the progression of diet induced nonalcoholic steatohepatitis (Oh et al., 2023). It also played a neuroprotective role in Alzheimer’s disease (AD) model mice by enhancing the phagocytosis of microglia against Aβ(Jo et al., 2022). However, whether GTIN is effective in treating pre-existing liver diseases and related complications has not been investigated.

In this study, we aimed to find therapeutic candidate for the treatment of chronic liver fibrosis patients with neurological symptoms. As carbon tetrachloride (CCl_4_) is a hepatotoxic drug which is commonly used to induce liver fibrosis in animal models (Rechnagel and Glende, 1973), we established a CCl_4_-induced mice model of liver fibrosis and tested the effect of gossypetin in treating pre-existing liver and brain pathologies.

## 2 Materials and Methods

### 2.1 Cell line and cell culture

LX-2 cells (Millipore Cat# SCC064, RRID: CVCL_5792) were cultured in 100 mm-cell culture dish or seeded at 10^5^ cells per ml in 6-Well dishes or 10^4^ cells per ml in glass coverslips placed in 24-well dishes, containing DMEM medium supplemented with 10% fetal bovine serum (FBS), 1% penicillin/streptomycin and grown in a 5% CO_2_ atmosphere at 37°C.

### 2.2 Cell experiment protocol

To determine whether GTIN can rescue LX-2 cells activation, we set up different processing groups:

H_2_O_2_ group: LX-2 cells were treated with 75 μmol H_2_O_2_ for 2 h (Chengdu Kelong Chemical Co., Ltd. 7722-84-1, H_2_O_2_ diluted with serum-free medium). Replaced fresh medium and continued to culture for 22 h (Cheng et al., 2015; Chen et al., 2022).

H_2_O_2_+GTIN group: LX-2 cells were treated with 75 μmol H_2_O_2_ (H_2_O_2_ diluted with serum-free medium) with 40 μmol GTIN for 2 h. Replaced fresh medium with 40 μmol GTIN and continued to culture for 22 h.

Control group: LX-2 cells were cultured with equal-volume DMEM for 2 h. Replaced fresh medium and continued to culture for 22 h.

### 2.3 Cell counting Kit-8

The LX-2 cells were seeded at 10^4^ cells in 96-Well dishes. The H_2_O_2_ group was treated with different concentration gradients of H_2_O_2_ solution (0, 50, 75, 100 μmol). The H_2_O_2_+GTIN group was treated in an equally graded H_2_O_2_ solution containing 40 μmol GTIN. The control group was treated with equal-volume DMEM. Two hours later, the H_2_O_2_ group and the control group were replaced with fresh medium. The H_2_O_2_+GTIN group was replaced with fresh medium containing 40 μmol GTIN. After 24 h, the proliferation-toxicity test of LX-2 cells was detected by Cell Counting Kit-8 (CCK-8, BS350A, Biosharp) according to the manufacturer’s instructions.

### 2.4 Western blot

Samples were lysed with ice-cold NP-40 lysis Buffer containing Phosphatase inhibitor cocktail A. After high-speed centrifugation, the protein supernatant was obtained, which was separated by SDS-PAGE polyacrylamide gel electrophoresis, transferred to polyvinylidene fluoride (PVDF) membrane, blocked by 5% nonfat-milk powder in Tris Buffered Saline with Tween 20 (TBST), incubated with primary antibody at 4°C overnight and the coupled secondary antibody at room temperature for 2 h. Finally, the image was developed by Enhanced chemiluminescence kit. The primary antibodies were α-Smooth Muscle Actin (α-SMA, Cell Signaling Technology Cat# 19245, RRID: AB_2734735), Phospho-p38 MAPK (Thr180/Tyr182) (P-p38 MAPK, Cell Signaling Technology Cat# 4511 (also 4511S), RRID: AB_2139682), Phospho-MKK3 (Ser189)/MKK6 (Ser207) (P-MKK3/MKK6, Cell Signaling Technology Cat# 12280, RRID: AB_2797868), Tumor protein 53 (p53, Cell Signaling Technology Cat# 2524, RRID:AB_331743) and glyceraldehyde-3-phosphate dehydrogenase (GAPDH, Abcam Cat# ab9485, RRID: AB_307275).

### 2.5 Immunocytochemistry

Cells growing on glass coverslips were fixed in 4% PFA solution at room temperature for 20 min, 0.1% Triton X-100 Phosphate Buffer Saline solution inducing cell membrane permeabilization, 1% Bovine Serum Albumin (BSA) Phosphate Buffer Saline with Tween 20 (PBST) solution blocked at room temperature. The primary antibody was incubated at 4°C overnight, abstersion. The coupled secondary antibody was incubated at room temperature for 1 h kept in dark place. Antifade Mounting Medium with 4′,6-diamidino-2-phenylindole (DAPI, P0131, Beyotime Biotechnology) incubated cell for 1 min. The primary antibody was Collagen type I alpha 1 chain (Col1a1, Cell Signaling Technology Cat# 72026, RRID: AB_2904565). Fluorescent secondary antibody was Goat Anti-Rabbit IgG H&L (Alexa Fluor ^®^ 488, Abcam Cat# ab150077, RRID: AB_2630356).

### 2.6 Animal

Eighteen SPF grade 5-week healthy wild-type male C57BL/6J mice, 25 ± 2 g, purchased from Gempharmatech, Jiangsu, without abnormal breathing, coarse hair, abnormal behavior or hunchback posture. They were kept in a clean room with 12 h sunshine, a temperature of about 25°C, and a relative humidity of about 40%–70%. They were free to eat water and forage for food. Follow-up experiments were conducted after 1 week of adaptation. After passing the experimental animal welfare ethical review of Chengdu Medical College, the mice were raised in the clean Laboratory Animal Room.

### 2.7 Experiments and preparation of specimens

The mice were randomly divided into 3 groups (*n* = 6 in each group). CCl_4_ (C805332, Macklin) was dissolved in Olive oil (O815210, Macklin), and mixed evenly. GTIN powder (B29179, Yuanyebio) was dissolved in Dimethyl sulfoxide (DMSO, D8371, Solarbio). Both CCl_4_ and GTIN were administered by intraperitoneal (i.p.) injection.

CCl_4_ group: Each mouse was injected with CCl_4_ solution for 6 weeks (CCl_4_: Oil = 1:4, 0.75 μ L/g, twice a week). In the last week, each mouse was injected with 300 mL of PBS daily. (Masuda et al., 2020; Guo et al., 2023; Xia et al., 2023).

CCl_4_+GTIN group: The injection time and method of CCl_4_ were the same as those of the CCl_4_ group. In the last week, each animal received a daily i.p. injection of GTIN (10 mg/kg). (Samant and Gupta, 2022; Oh et al., 2023).

Control group: The control group was injected with equal volume PBS.

### 2.8 Behavior detection

#### 2.8.1 Elevated plus maze

Elevated plus maze is composed of two open arms and two closed arms in the shape of a cross. The arms are 5 cm wide and 35 cm long, the closed arms have a 15 cm high wall, and the maze sits 40–55 cm above the ground. The mouse was gently placed in the central area of the cross, and the movement path of the mouse was tracked by ANY-MAZE software for 5 min. The number of open arm entries (OE), closed arm entries (CE) were recorded. The percentage of numbers entering the open arm (OE%) = OE/(OE + CE) ×100%.

#### 2.8.2 Tail suspension test

Medical tape was used to quickly secure the tip of the tail at the 1 cm mark, hanging the mouse upside down from a sturdy rod, with the height from the tip of the mouse’s tail to the platform being approximately 30 cm. The behavior of the mouse attempting to stand up or struggle is recorded as struggling state, while immobility state is the state when the mouse ceases struggling and maintains a vertical inverted position or exhibits swings due to inertia. The total test duration is 6 min, with the immobility time of the mouse recorded during the latter 4 min.

#### 2.8.3 New object recognition

On the first day of the adaptation period, the mice were placed in the open field for 10 min. During the familiarization period on the second day, the same objects were placed in two adjacent corners of the open field, the objects were 10 cm away from the side wall of the open field, and the mice were placed at an equal distance from the objects with their backs to the objects, within 2–3 cm of the objects as the exploration range, with a total duration of 5 min. The exploration times, time and distance of the mice in each object were recorded through ANY-MAZE software. On the third day of testing, one of the objects was replaced with a new object and placed in the same position in the open field, and the rest of the operations were the same as on the second day. Cognitive index = Time spent exploring new objects/time spent exploring old objects × 100%.

#### 2.8.4 Y-maze test

Prepare a Y-maze consisting of 3 arms of equal length, noted as A arm, B arm, and C arm, with an Angle of 120 ° between each arm. The inner wall of the maze was all white. Mice were placed at the end of arm A and allowed to explore freely for 8 min. The total number of entry times and the numbers of rotation of were recorded. The standard for mice entering an arm is for all four limbs to be fully inside. Consecutive entry into three different arms is recorded as one rotation. Alternation (%) = number of rotations/(total number of entry arm - 2) ×100%.

#### 2.8.5 Pole test

Prepare a vertical pole with gauze wrapped around the surface to allow the mouse a firm grip. The mice were placed on top of the pole and recorded how long it took them to reach the bottom of the pole with both back feet touching the ground. Each mouse was repeated 5 times and trained 2 times before formal detection.

#### 2.8.6 Open field test

The open field is a 40 cm square in length, width and height. The mice were gently placed in the center of the open field, and the movement were tracked through ANY-MAZE software for a total duration of 10 min, and the total distance and average speed were recorded.

### 2.9 Animal handling and specimen preparation

The mice were anesthetized with 4% chloral hydrate (100 mL/kg i.p.). After cutting off the whiskers, the eyeballs were pressed, tweezed, quickly removed and the blood was collected into 15 mL centrifuge tubes, placed at room temperature for 30 min, and centrifuged at 4°C (2000 rpm, 20 min). The serum was carefully pipetted into 1.5 mL tubes, marked, and stored at −20°C for subsequent serological assays.

After blood collection, each mouse was immediately injected with pre-cooled PBS solution through the heart and continued with pre-cooled 4% paraformaldehyde (PFA) until liver discoloration. The brain and liver were gently extracted and fixed in PFA overnight and were washed with water for 6 h and stored in 75% ethanol.

### 2.10 Histomorphology

#### 2.10.1 Tissue processing

After fixation with 4% PFA, the brain and liver were dehydrated with gradient ethanol (75%, 85%, 95%, 100%), xylene, embedded with paraffin, and sliced in wax blocks (liver Section 6 μm thick, brain Section 4 μm thick).

Paraffin sections of liver were stained with Hematoxylin and Eosin (H&E, CS700, CS701, CS702, Aligent), Modified Sirius Red Stain Kit (No Picric Acid) (G1472, Solarbio).

#### 2.10.2 Immunohistochemical staining

Paraffin sections of liver were dewaxed into water and heat-induced antigen retrieval method. After cooling, the tissues were blocked from endogenous peroxidase activity and incubated overnight with primary antibody. The slices were washed with PBS and incubated with enzyme-labeled sheep anti-rabbit IgG polymer or enzyme-labeled sheep anti-mouse IgG polymer for 30 min followed by freshly prepared diaminobenzidine (DAB) solution. Finally, the sections were subjected to gradient alcohol dehydration, xylene transparent, neutral gum seal. The primary antibodies were Col1a1 (Cell Signaling Technology Cat# 72026, RRID: AB_2904565), α-SMA (Cell Signaling Technology Cat# 19245, RRID: AB_2734735), Phospho-p38 MAPK (Thr180/Tyr182) (P-p38 MAPK, Cell Signaling Technology Cat# 4511 (also 4511S), RRID: AB_2139682), Phospho-MKK3 (Ser189)/MKK6 (Ser207) (P-MKK3/MKK6, Cell Signaling Technology Cat# 12280, RRID: AB_2797868), Tumor protein 53 (p53, Cell Signaling Technology Cat# 2524, RRID:AB_331743), CD68/SR-D1 (CD68, Novus Cat# NB100-683, RRID: AB_2074852), Complement component 3 receptor 3 subunit (CD11b, Thermo Fisher Scientific Cat# MA5-17857, RRID: AB_2539241). Appropriate paraffin sections of brain tissue were selected for IHC staining. The primary antibodies were Glial fibrillary acidic protein (GFAP, Millipore Cat# MAB360, RRID: AB_11212597), Ionized calcium binding Adapter molecule 1 (Iba1, Abcam Cat# ab178846, RRID: AB_2636859).

In each group, 5 mice livers and 3 mice brains were used for serial sections of the regions of interest. IHC staining was performed, and images were taken with optical microscope (BX63, Olympus) or Digital Pathology Slide Scanner (KF-FL-005, KFBIO). When taking high magnification images of the liver, a lobule with a central vein in the center was shown. The images were imported to FIJI software (v2.3.0, RRID:SCR_002285, United States). The area and integrated density of the positive region of the whole image was measured. To select the positive region, we used the software’s automatic threshold followed by manually adjusting the lower and upper thresholds in combination with the color depth and position of the original image to completely cover the positive area but not the background and negative area. The same protein used the same interval values while different proteins correspond to different lower and upper thresholds. The gray value was converted to optical density (OD) value and the integrated density was obtained. The integrated density of Con group was normalized, and the relative Integrated density of CCl_4_ group and CCl_4_+GTIN group was calculated.

Sections in the same brain regions were selected for staining and comparative analysis. Similar statistical methods were used for analyzing Iba1 and GFAP in Amygdala, Striatum and Cortex by measuring the percentage of positive area (% area). For the hippocampus CA1 region, we only analyze the area within the dashed line. We also manually counted the numbers of positive cells with cell bodies within the target brain regions. Serial section images were used for counting and the average numbers were used as the value for 1 mouse. The cell density was expressed in terms of the number of cells per square millimeter (cells/mm^2^).

#### 2.10.3 Nissl staining

Appropriate brain tissue sections were dewaxed, hydrated and stained with Nissl stain kit (G1430, Solarbio) according to the instructions. To put it simply, added the section into Crysal Violet Stain, incubated in 56°C incubator for 1 h, rinsed with deionized water and differentiated in Nith Differentiation Solution for 2 min. The purple staining showed Nissl bodies, representing neurons; the background was colorless or light blue.

### 2.11 Detection of serum ALT and AST activity

Serum alanine aminotransferase (ALT) and aspartate transaminase (AST) activity was detected using Mouse ALT ELISA Kit (MM-0260M1, Meimian) and Mouse AST ELISA Kit (MM-44384M1, Meimian) according to the standardized procedures suggested by the manufacturer. Both ALT and AST were shown in units per liter (U/L).

### 2.12 Real-time PCR analysis

Total RNA was extracted from fresh liver tissues by Trizol Reagent (T9424, Sigma). Reverse transcription was performed using PrimeScript™ RT reagent Kit with gDNA Eraser (RR047A, Takara), according to the manufacturer’s instructions. qPCR with TB Green^®^ Premix Ex Taq™ II (RR820A, Takara) and primers, according to the manufacturer’s instructions. *GAPDH* was used as the internal reference. 2^ (- ∆∆ Cq) was used for statistical analysis. The sequences of primers are shown in Table 1.

**TABLE 1:** Primer sequences.

Gene	Forward primers (5′-3′)	Reverse Primers (5′-3′)
*GAPDH*	AGG​TCG​GTG​TGA​ACG​GAT​TTG	TGT​AGA​CCA​TGT​AGT​TGA​GGT​CA
*IL-1α*	CGA​AGA​CTA​CAG​TTC​TGC​CAT​T	GAC​GTT​TCA​GAG​GTT​CTC​AGA​G
*IL-1β*	GCA​ACT​GTT​CCT​GAA​CTC​AAC​T	ATC​TTT​TGG​GGT​CCG​TCA​ACT
*IL-6*	TAG​TCC​TTC​CTA​CCC​CAA​TTT​CC	TTG​GTC​CTT​AGC​CAC​TCC​TTC
*IL-10*	TGC​ACT​ACC​AAA​GCC​ACA​AG	TCA​GTA​AGA​GCA​GGC​AGC​AT
*TNF-α*	CAT​CTT​CTC​AAA​ATT​CGA​GTG​ACA​A	TGG​GAG​TAG​ACA​AGG​TAC​AAC​CC

### 2.13 Statistical analysis

Quantitative data were statistically analyzed by GraphPad prime (v9.1.1, RRID:SCR_002798, United States). All numerical variables are expressed as (Means ± SEM). Group differences were analyzed with One-way ANOVA. *p* < 0.05 was considered a statistical difference.

## 3 Result

### 3.1 GTIN alleviates H_2_O_2_-induced activation of LX-2 cells

**FIGURE 1:**
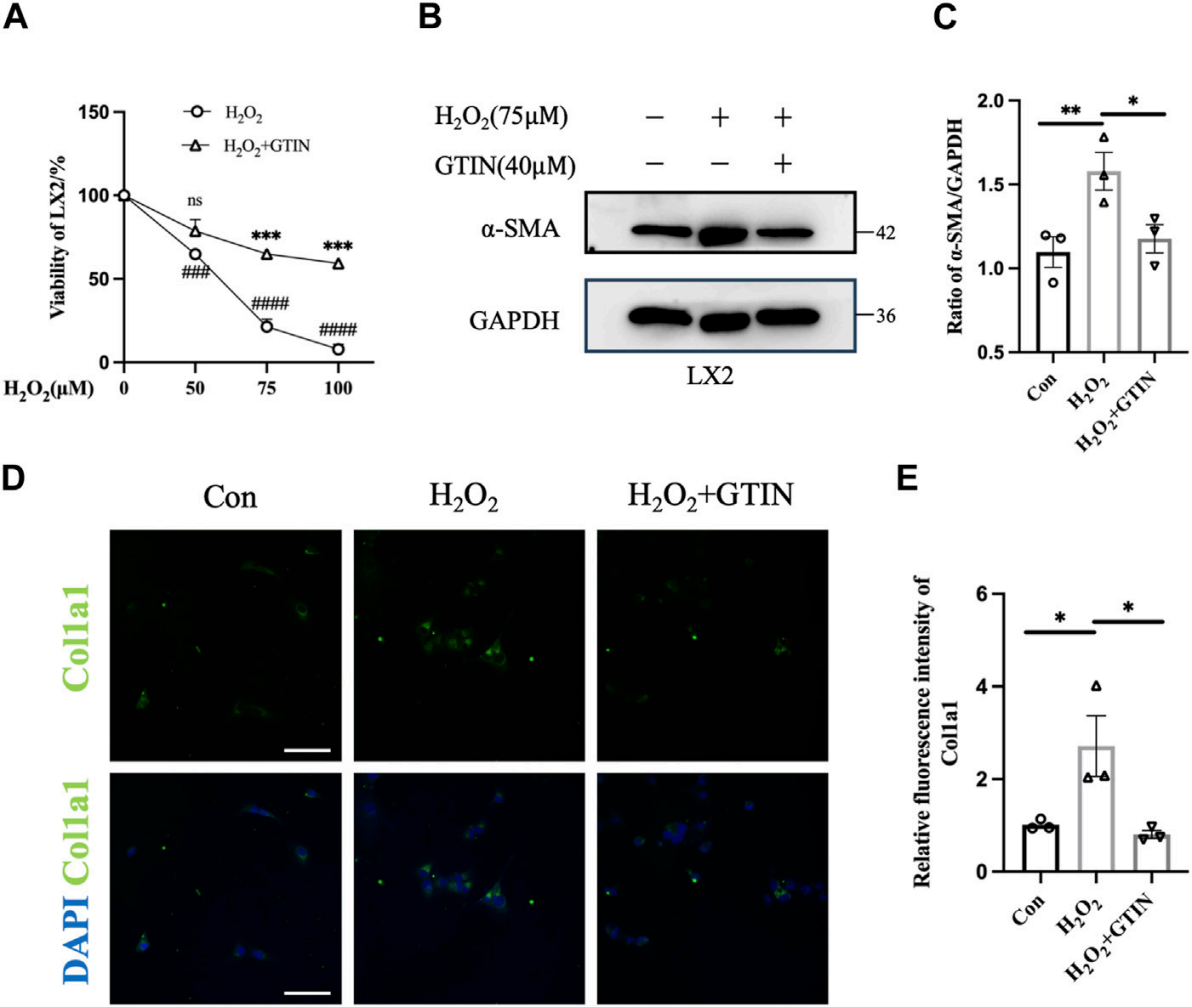
GTIN partially inhibited LX-2 cells activation induced by oxidative stress. **(A)** Viability of LX-2 cells after treating with different concentrations of H_2_O_2_ with or without 40 μmol GTIN by cell counting kit 8 (CCK8) (*n* = 3). **(B, C)** Western blotting and the statistical analysis of relative expression of α-SMA in LX-2 cells treated with 75 μmol H_2_O_2_ with or without 40 μmol GTIN (*n* = 3). **(D, E)** Immunofluorescence staining and the statistical analysis of Col1a1 in LX-2 cells (*n* = 3). *p < 0.05 compared to H_2_O_2_ group, **p < 0.01 compared to H_2_O_2_ group, ***p < 0.001 compared to CCl_4_ group, ns means no significant difference compared to H_2_O_2_ group, ###*p* < 0.001 compared to 0 μmol H_2_O_2_, ####*p* < 0.0001 compared to 0 μmol H_2_O_2_, Scale bar: 100 μm.

As oxidative stress plays an important role in the development of liver fibrosis (Singal et al., 2011), we used hydrogen peroxide to stimulate LX-2 cells to mimic liver injuries caused by oxidative stress. Increased H_2_O_2_ concentrations lead to decreased LX-2 viability, which can be rescued by 40 μmol GTIN (Figure 1A). The expression of α-SMA protein was increased upon H_2_O_2_ treatment (Figures 1B,C), indicating that LX-2 cells were activated by oxidative stress. The use of GTIN restored α-SMA protein expression (Figures 1B,C). Similarly, immunofluorescence revealed that increased Col1a1 expression in H_2_O_2_-stressed LX-2 cells was abolished by GTIN treatment (Figures 1D,E). These results indicated that GTIN could suppress the LX-2 activation induced by oxidative stress.

### 3.2 Short-term administration of GTIN ameliorates CCl_4_-induced liver damage and associated neurological symptoms in mice

**FIGURE 2:**
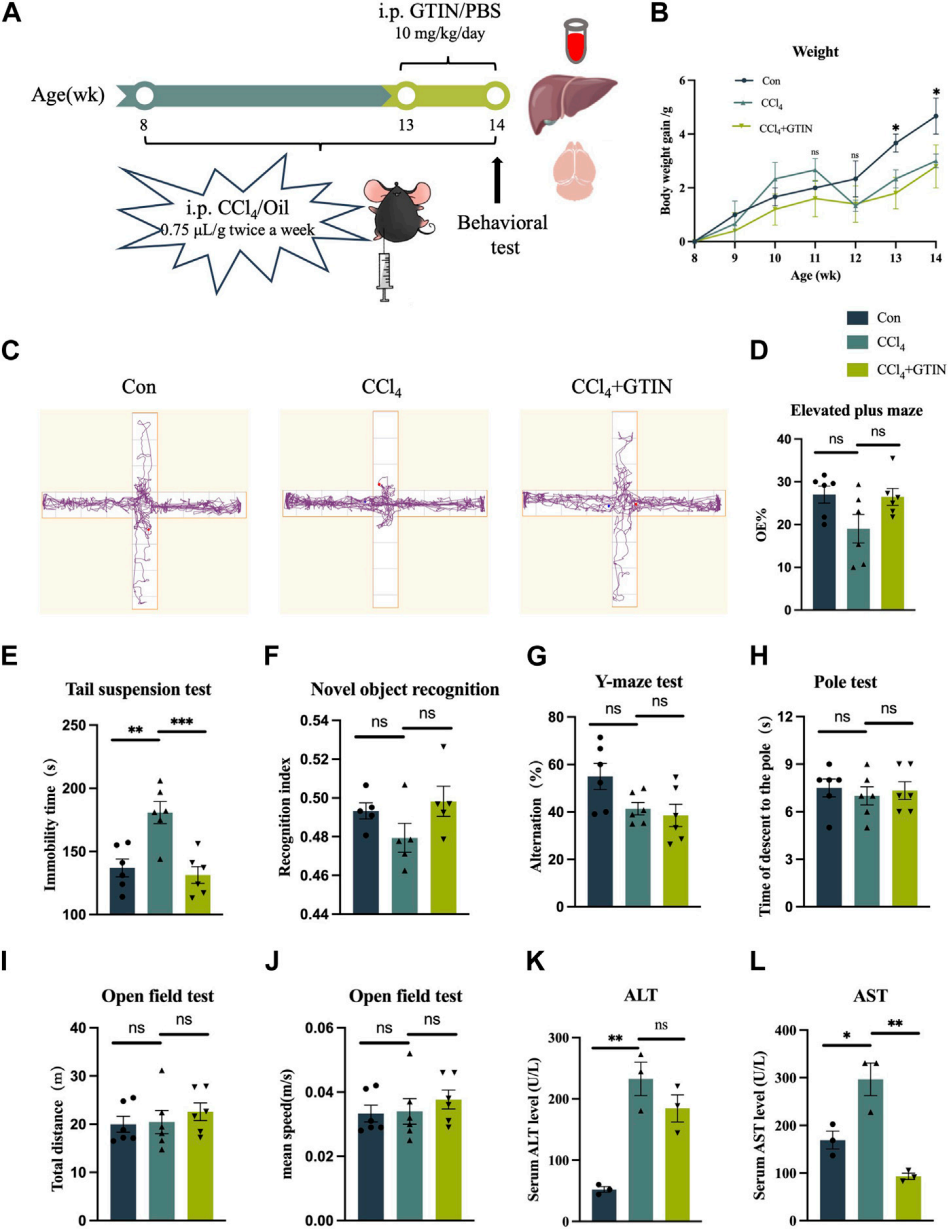
GTIN ameliorated CCl_4_-induced liver function impairment and behavioral change in C57BL/6J male mice. **(A)** Schematic diagram of hepatic fibrosis mouse model establishment and treatment process, including CCl_4_ and GTIN injection dose and time. **(B)** Weight changes in mice with liver fibrosis treated with or without GTIN. **(C)** Motion track diagram of mouse Elevated plus maze experiment. Quantitative analysis of **(D)** the percentage of numbers entering the open arm in Elevated plus maze experiment, **(E)** immobility time in Tail suspension test, **(F)** recognition index in New object recognition experiment, **(G)** spontaneous alternation rate in Y-maze experiment, **(H)** time of climbing to the ground in the Pole test, **(I)** total distance and **(J)** mean speed in Open field test in each group of mice. (new object recognition experiment *n* = 5, other behavior test *n* = 6). **(K, L)** Quantitative analysis of serum AST and ALT levels in each group of mice by ELISA (*n* = 3). *p < 0.05 compared to CCl_4_ group, **p < 0.01 compared to CCl_4_ group, ***p < 0.001 compared to CCl_4_ group, ns means no significant difference compared to CCl_4_ group.

To clarify the effect of GTIN on pre-existing liver fibrosis, we established a liver fibrosis model by intraperitoneal injection CCl_4_ in male C57BL/6J mice for 6 weeks (Figure 2A). The CCl_4_+GTIN group received intraperitoneal injections of GTIN once a day in the last week of CCl_4_ injection. Mice injected with CCl_4_ became lethargic, and their hair gradually began to lose its luster. The average weight gain of mice in CCl_4_ group was lower than those in the control group from the fifth week to the end of the experiment (Figure 2B). At the end of the 6-week course of the experiment, behavioral tests were performed on the three groups of mice. In elevated plus maze, CCl_4_ decreased the percentage of visits to the open arm and GTIN ameliorated this (Figures 2C,D). Tail suspension experiment showed a significantly increased immobile time of mice treated with CCl_4_, and GTIN rescued this increase (Figure 2E). These results suggest that anxiety and depression like behavior induced by CCl_4_ could be rescued by short-term GTIN treatment. In the new object recognition experiment, mice in the CCl_4_ group spent less time exploring new objects and had lower recognitive indexes, which were improved by GTIN (Figure 2F). The lack of statistical significance may be because cognition-related experiments have greater variations within the group and require more mice in the experiments. Likewise, the spontaneous alternating rate of Y-mazes test in CCl_4_ and CCl_4_+GTIN mice decreased compared to the control group (Figure 2G). On the contrary, CCl_4_ does not influence motor performance according to open-field and pole test (Figures 2H–J). After the behavioral experiment, mice were euthanized, serum, liver tissues and brain tissues were collected for subsequent analysis (Figure 2A). Compared with the control group, serum aspartate aminotransferase (AST) and alanine aminotransferase (ALT) enzyme activities in CCl_4_ group dramatically increased, indicating impairment of liver function (Figures 2K,L). Short-term GTIN treatment reduced both AST and ALT enzyme activity, though the decrease of ALT is not statistically significant (Figures 2K,L). These results suggested that GTIN could alleviate CCl_4_-induced behavioral change and liver function decline in mice.

### 3.3 GTIN alleviates pre-existing liver fibrosis induced by CCl_4_

**FIGURE 3:**
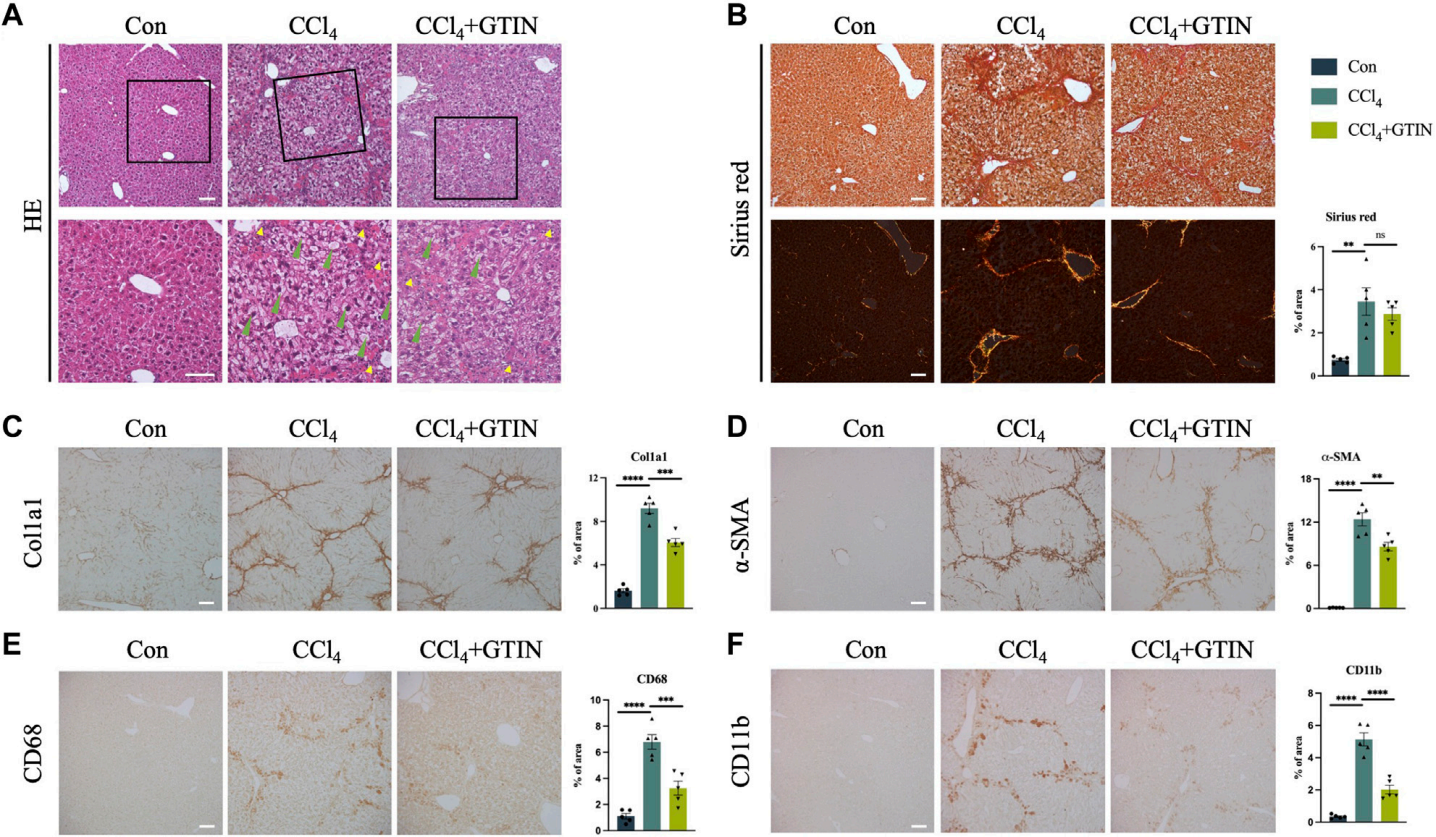
GTIN rescued CCl_4_-induced pre-existing liver fibrosis in C57BL/6J male mice. **(A)** Representative sections of liver HE stains. Green arrows indicate balloon-like hepatocytes. Yellow arrows indicate infiltration of inflammatory cells in the portal area. Representative sections and quantitative analysis of positive area of **(B)** Sirius red staining labeled fiber intervals, IHC staining including **(C)** Col1a1 labeled collagen fibroplasia, **(D)** α-SMA labeled activated HSCs, KCs markers including **(E)** CD68 and **(F)** CD11b in each group of mice (*n* = 5). **p < 0.01 compared to CCl_4_ group, ***p < 0.001 compared to CCl_4_ group, ****p < 0.0001 compared to CCl_4_ group, ns means no significant difference compared to CCl_4_ group, Scale bar: 100 μm.

We examined the appearance of the whole liver. The livers from the control group were smooth with soft texture and reddish-brown color, while the livers from CCl_4_ and GTIN groups were enlarged with mottled surface and granulated texture. Hematoxylin and Eosin (HE) staining (Figure 3A) showed that the control group has normal liver lobular structure. In CCl_4_ group, the structure of hepatic lobules was preserved, but the volume of hepatic cells in the lobules was increased, hepatocytes were crowded, hepatic sinuses were narrow, red fine particles could be seen in the cytoplasm, and cell edema appeared. There was more inflammatory cell infiltration in the sink area and a small amount of fibrous tissue hyperplasia in the interlobular. After short-term treatment with GTIN, the degree of hepatocyte edema and inflammatory cell infiltration was reduced. Sirius red staining showed that these proliferating collagen fibers were type 1 collagen fibers with a strong orange-yellow color (Figure 3B). GTIN treatment decreased Sirius red positive collagen fiber content but not statistically significant (Figure 3B). We also use Col1a1 as another marker of liver fibrosis and observed solid alleviation of liver fibrosis upon GTIN treatment (Figure 3C). IHC of α-smooth muscle actin (α-SMA), a marker of HSCs activation, got similar results, indicating that GTIN effectively suppressed the activation of HSCs (Figure 3D). Kupffer cells (KCs) directly regulate the activation of HSCs and promote the development of liver fibrosis. KCs differentiate into different subtypes under different stimuli and have different roles (Wang et al., 2021). The two most common subtypes are CD68-positive KCs with phagocytic function, and CD11b-positive with cytokine secretion function (Kinoshita et al., 2010; Wen et al., 2021; Kulle et al., 2022). Therefore, we examined these two types of KCs in different groups. In CCl_4_ group, both were significantly increased, located around the hepatic lobules (Figures 3E, F). GTIN reduced both CD68 and CD11b positive KCs (Figures 3E, F).

Collectively, our results suggest that short-term of GTIN treatment is sufficient to alleviate pre-existing liver fibrosis induced by CCl_4_.

### 3.4 GTIN restores CCl_4_-induced activation of MKK3/6-p38 MAPK signaling and liver inflammation

**FIGURE 4:**
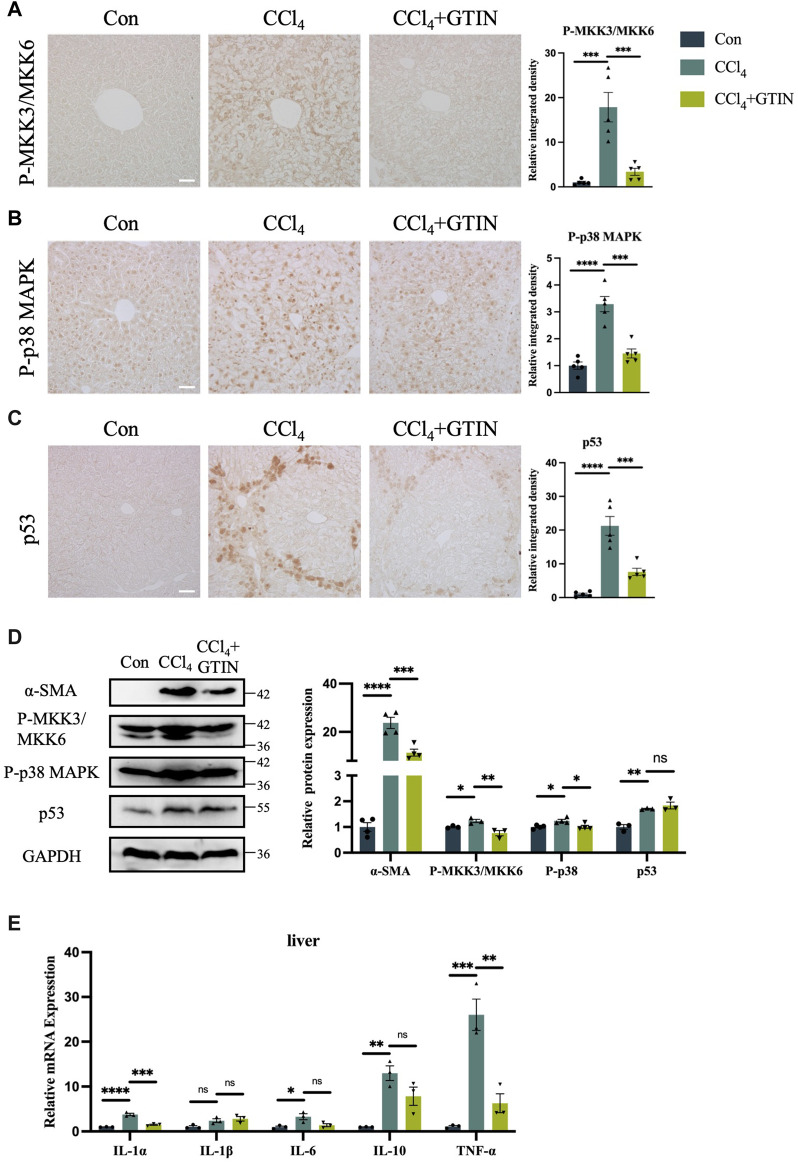
CCl_4_ induced liver MKK3/6-p38 MAPK activation and inflammation can be effectively suppressed by GTIN. The experimental method is also shown in Figure 2A. **(A–C)** Liver representative sections and quantitative analysis of P-MKK3/MKK6, P-p38 MAPK, p53 protein IHC staining in each group of mice (*n* = 5). **(D)** Western blot results and quantitative analysis of α-SMA (*n* = 4), P-MKK3/MKK6 (n = 3), P-p38 MAPK (*n* = 4) and p53 (*n* = 3) proteins in mice liver. **(E)** IL-1α, IL-1β, IL-6, IL-10, TNF-α mRNA expression in each group of mice liver (*n* = 3). *p < 0.05 compared to CCl_4_ group, **p < 0.01 compared to CCl_4_ group, ***p < 0.001 compared to CCl_4_ group, ****p < 0.0001 compared to CCl_4_ group, ns means no significant difference compared to CCl_4_ group, Scale bar: 50 μm.

p38 MAPK signaling plays an important role in the development of liver diseases (Cicuéndez et al., 2021). Upon activation by Ser189/Thr222 phosphorylation of MKK3 and Ser207/Thr211 of MKK6, they activate downstream p38 MAPK by phosphorylation of Thr180 and Tyr182 of p38α (Han et al., 1996; Raingeaud et al., 1996; Ge et al., 2002). As GTIN was shown to be an inhibitor of MKK3/6 signaling in esophageal cancer (Xie et al., 2019), we investigated whether GTIN could regulate MKK3/6-p38 MAPK signaling in our mice model. The expression of P-MKK3/MKK6 and P-p38 MAPK in CCl_4_ group was significantly increased compared to control, especially in the cytoplasm of hepatocytes surrounding the hepatic central vein (Figures 4A,B). GTIN treatment decreased CCl_4_-induced activation of P-MKK3/MKK6 and P-p38 MAPK (Figures 4A,B). Since p38 MAPK could regulate cell survival and cell death through p53, we detected the expression of p53 in the liver of our mice. As shown in Figure 4C, the expression of p53 protein was significantly increased in CCl_4_ group, indicating that apoptosis occurred, whereas GTIN could suppress p53 activation by CCl_4_. WB results showed that the expression of α-SMA, P-MKK3/MKK6, P-p38 MAPK, p53 in liver was significantly upregulated by CCl_4_, and the expression of α-SMA, P-MKK3/MKK6, P-p38 MAPK could be rescued by GTIN (Figure 4D). During the immune response, p38α regulates the phosphorylation of various enzymes, transcription factors, regulatory proteins, and DNA/RNA binding proteins, which can directly lead to the production and secretion of inflammatory factors (Canovas and Nebreda, 2021), thus we examined the expression of cytokines in liver tissue. IL-1α, IL-6, IL-10, and TNF-α were all upregulated due to CCl_4_ stimulation, while the IL-1α and TNF-α mRNA level were significantly reduced after GTIN treatment (Figure 4E).

These results suggest that GTIN suppresses CCl_4_-induced activation of liver MKK3/6-p38 MAPK signaling pathway, leading to ameliorated p53 overactivation and reduced hepatic inflammation.

### 3.5 GTIN alleviates hippocampal neuroinflammation induced by CCl_4_

**FIGURE 5:**
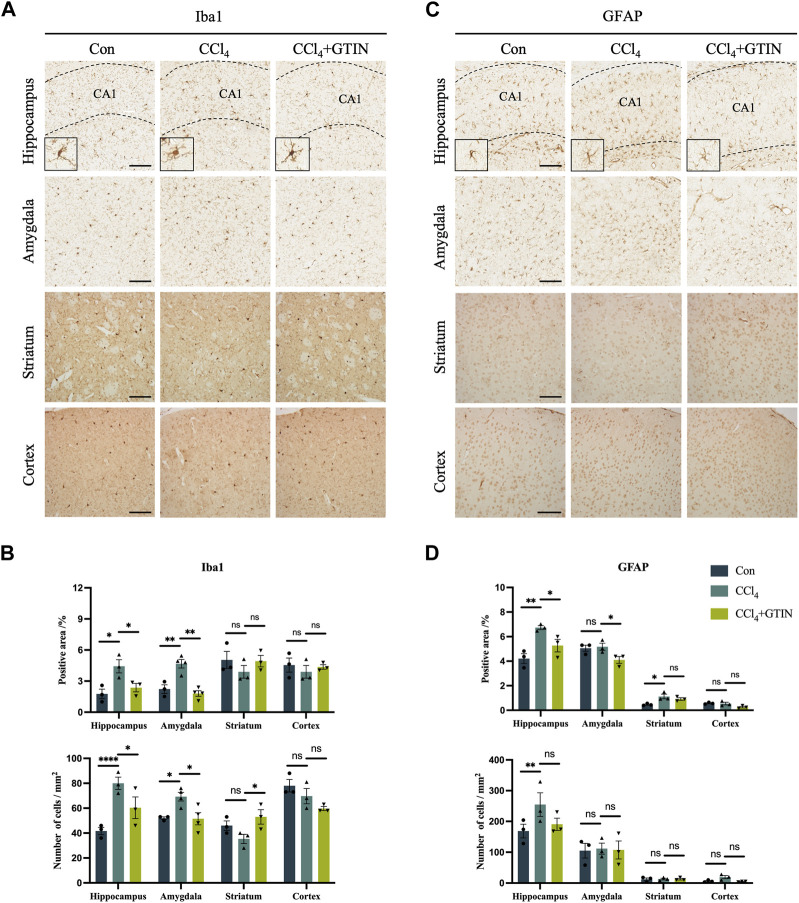
Liver fibrosis induced by CCl_4_ causes differential microglia activation and astrogliosis in different brain regions. **(A, C)** Representative brain sections and of IHC staining of Iba1 (a marker of microglia) and GFAP (a marker of astrocyte) in hippocampus, amygdala, cortex or striatum in those hepatic fibrosis model mice treated with or without GTIN. The morphology of microglia in high magnification field is shown in the bottom left corner. **(B, D)** Quantitative analysis of percentage positive area and positive cell number of Iba1 and GFAP in each brain area. (Iba1 in amygdala CCl_4_, GTIN *n* = 4, other group *n* = 3). *p < 0.05 compared to CCl_4_ group, **p < 0.01 compared to CCl_4_ group, ***p < 0.001 compared to CCl_4_ group, ns means no significant difference compared to CCl_4_ group, Scale bar: 100 μm.

As an organ that plays important roles in metabolism and detoxification, chronic liver disease often influences other systems including the nervous system. A plethora of clinical studies revealed that chronic liver diseases were associated with neurological symptoms including anxiety, depression, and accelerated cognitive decline (Filipović et al., 2018; Labenz et al., 2020; Jiang et al., 2023). Thus, we investigated whether there were pathological alterations in the brain of hepatic fibrosis mice. IHC staining of Iba1 showed that compared with the control group, the number and area of Iba1-positive microglia in hippocampal (CA1) region and amygdala region in CCl_4_ group was significantly increased (Figures 5A,B). CCl_4_ induction led to the increase in the volume of microglia cells in the hippocampus and the decrease in the number of processes, called reactive microglia. After one-week of GTIN administration, microglia returned to a resting state (Figure 5A). The number and morphology of microglia in striatum and cortex did not change significantly (Figures 5A,B). Astrocytes in the same brain area were detected by GFAP IHC staining. Compared with the control group, the number and area of GFAP-positive astrocytes in hippocampal (CA1) and striatum of mice in CCl_4_ group increased (Figures 5C,D). Increased and elongated astrocyte branches were also observed in the hippocampus, indicating occurrence of astrocytes in this area (Figure 5C). GTIN treatment not only reduced the number of astrocytes but also rescued the morphological change of astrocytes in the hippocampus (Figures 5C,D). In the amygdala and cortex, the number and morphology of astrocytes did not change significantly (Figures 5C,D).

Collectively, our results show that short-term GTIN treatment ameliorates neuroinflammation in the hippocampus of CCl_4_-treated mice, as indicated by decreased microglial and astrocyte activation.

### 3.6 No neuronal loss was observed in CCl_4_-treated mice brain

**FIGURE 6:**
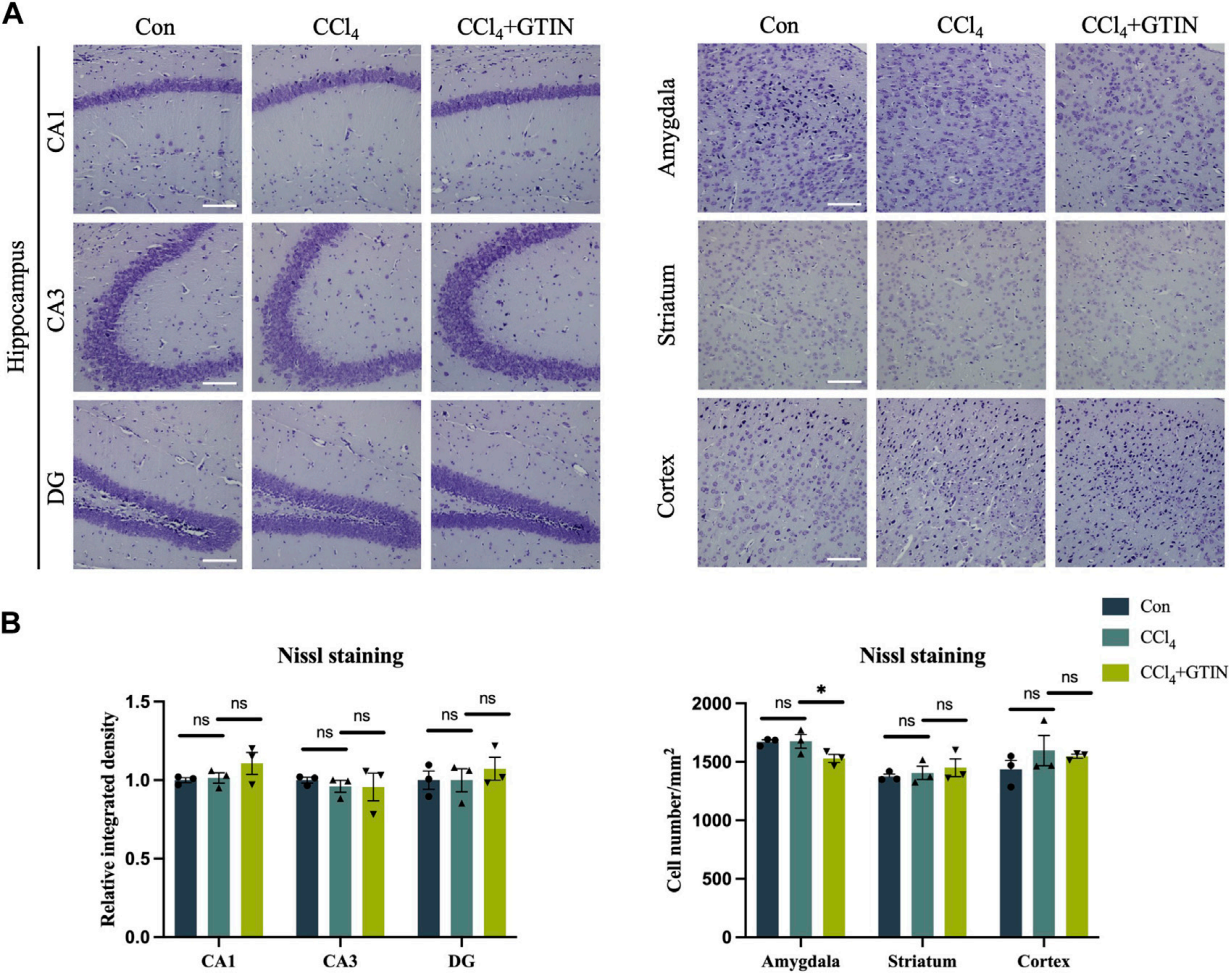
Liver inflammation did not cause neuronal loss in above brain regions. Representative brain sections and quantitative analysis of Nissl staining in **(A, B)** hippocampus, amygdala, striatum or cortex in those hepatic fibrosis model mice treated with or without GTIN (*n* = 3). *p < 0.05 compared to CCl_4_ group, ns means no significant difference compared to CCl_4_ group, Scale bar: 100 μm.

We also detected the number of neurons in brain regions including hippocampus, amygdala, striatum, and cortex using Nissl staining. No difference of Nissl bodies in these brain regions of control and CCl_4_-treated mice was observed (Figures 6A,B). Thus, CCl_4_ treatment did not cause neuronal loss in the above brain area.

## 4 Discussion

In this research, we found that pathological change in the liver could induce neuroinflammation in certain regions of the brain, and brief administration of GTIN is sufficient to ameliorate both liver fibrosis and neuroinflammation. Although previous studies have revealed the hepatoprotective effect of GTIN, it was administered at the beginning or in the whole course of the experiment (Chen et al., 2013; Khan et al., 2015; Oh et al., 2023). Thus, whether GTIN ameliorates pre-existing liver diseases is unknown. In our experiments, we administered GTIN only in the last week of a 6-week consecutive CCl_4_ treatment. As liver fibrosis has been induced when GTIN was used, we could evaluate whether GTIN was useful in treating pre-existing liver fibrosis. We found that short-term administration of GTIN was sufficient to rescue impaired liver function and alleviate pre-existing liver fibrosis induced by CCl_4_. We also verified that GTIN is a potent inhibitor of MKK3/6-p38 MAPK pathway in our model of liver damage. The downstream targets of p38 MAPK that led to aggravation of liver fibrosis were also suppressed by GTIN. This discovery shows the potential of this flavonoid in treating liver fibrosis, not only delaying its development.

As the liver is a vital organ that plays a crucial role in metabolism and detoxification, impairment of its function may influence other organs including the brain (García-Martínez and Córdoba, 2011; Schwendimann and Minagar, 2017). Chronic liver diseases led to accelerated cognitive decline and reduced grey matter in certain brain regions (Labenz et al., 2020; Jiang et al., 2023; Xu et al., 2023). Hippocampus, the brain region responsible for learning and memory (Lisman et al., 2017; Yavas et al., 2019), is among the most affected brain regions of chronic liver disease patients. Thus, another consideration is to find therapeutic candidates that work in both liver and brain. We focused on GTIN because previous studies have revealed its neuroprotective role in a model mouse of AD (Jo et al., 2022). Although we observed no neuronal loss in our mice, there was indeed astrogliosis and activated microglia in certain brain regions of the CCl_4_-treated mice, and brief GTIN treatment is sufficient to alleviate hippocampal neuroinflammation induced by liver fibrosis. Considering that prolonged neuroinflammation in the hippocampus may contribute to advanced pathological changes that related to cognitive decline, the anti-neuroinflammation character of GTIN makes it a potential therapeutic option to counteract accelerated cognitive decline induced by chronic liver diseases.

To sum up, our results suggest that GTIN is an effective therapeutic candidate for treating liver fibrosis. Short term of administration is sufficient to restore liver function and ameliorate inflammation in animals with pre-existing liver fibrosis. GTIN also shows the potential to reduce neuroinflammation in certain regions of the brain, which may contribute to the neurological symptoms if it persists. GTIN’s capability in targeting liver-brain axis makes it a promising therapeutic candidate for chronic liver disease patients.

## Data Availability

The original contributions presented in the study are included in the article, further inquiries can be directed to the corresponding authors.

## References

[B1] Butterworth R. F. (2013). The liver-brain axis in liver failure: neuroinflammation and encephalopathy. Nat. Rev. Gastroenterol. Hepatol. 10 (9), 522–528. 10.1038/nrgastro.2013.99 23817325

[B2] Canovas B. Nebreda A. R. (2021). Diversity and versatility of p38 kinase signalling in health and disease. Nat. Rev. Mol. Cell Biol. 22 (5), 346–366. 10.1038/s41580-020-00322-w 33504982 PMC7838852

[B3] Chang L. Karin M. (2001). Mammalian MAP kinase signalling cascades. Nature 410 (6824), 37–40. 10.1038/35065000 11242034

[B4] Chen J. Guo Q. Chen Q. Chen Y. Chen D. Chen Z. (2022). Interleukin 10 inhibits oxidative stress-induced autophagosome formation in hepatic stellate cells by activating the mTOR-STAT3 pathway. Exp. Cell Res. 411 (2), 113001. 10.1016/j.yexcr.2021.113001 34973945

[B5] Chen J. H. Tsai C. W. Wang C. P. Lin H. H. (2013). Anti-atherosclerotic potential of gossypetin via inhibiting LDL oxidation and foam cell formation. Toxicol. Appl. Pharmacol. 272 (2), 313–324. 10.1016/j.taap.2013.06.027 23845592

[B6] Cheng J. Wang M. Ma H. Li H. Ren J. Wang R. (2015). Adiponectin inhibits oxidative stress and modulates TGF-b1 and COL-1 expression via the AMPK pathway in HSC-T6 cells. Zhonghua Gan Zang Bing Za Zhi 23 (1), 69–72. 10.3760/cma.j.issn.1007-3418.2015.01.016 25751391 PMC12770590

[B7] Choisy-Rossi C. Yonish-Rouach E. (1998). Apoptosis and the cell cycle: the p53 connection. Cell Death Differ. 5 (2), 129–131. 10.1038/sj.cdd.4400339 10200456

[B8] Cicuéndez B. Ruiz-Garrido I. Mora A. Sabio G. (2021). Stress kinases in the development of liver steatosis and hepatocellular carcinoma. Mol. Metab. 50, 101190. 10.1016/j.molmet.2021.101190 33588102 PMC8324677

[B9] Filipović B. Marković O. Đurić V. Filipović B. (2018). Cognitive changes and brain volume reduction in patients with nonalcoholic fatty liver disease. Can. J. Gastroenterol. Hepatol. 2018, 9638797. 10.1155/2018/9638797 29682494 PMC5848059

[B10] Gan C. Cai Q. Tang C. Gao J. (2022). Inflammasomes and pyroptosis of liver cells in liver fibrosis. Front. Immunol. 13, 896473. 10.3389/fimmu.2022.896473 35707547 PMC9189314

[B11] García-Martínez R. Córdoba J. (2011). Acute-on-chronic liver failure: the brain. Curr. Opin. Crit. Care 17 (2), 177–183. 10.1097/MCC.0b013e328344b37e 21346567

[B12] Ge B. Gram H. Di Padova F. Huang B. New L. Ulevitch R. J. (2002). MAPKK-independent activation of p38alpha mediated by TAB1-dependent autophosphorylation of p38alpha. Science 295 (5558), 1291–1294. 10.1126/science.1067289 11847341

[B13] González-Terán B. Matesanz N. Nikolic I. Verdugo M. A. Sreeramkumar V. Hernández-Cosido L. (2016). p38γ and p38δ reprogram liver metabolism by modulating neutrophil infiltration. Embo J. 35 (5), 536–552. 10.15252/embj.201591857 26843485 PMC4772851

[B14] Guo P. C. Zuo J. Huang K. K. Lai G. Y. Zhang X. An J. (2023). Cell atlas of CCl (4)-induced progressive liver fibrosis reveals stage-specific responses. Zool. Res. 44 (3), 451–466. 10.24272/j.issn.2095-8137.2023.031 36994536 PMC10236302

[B15] Hafner A. Bulyk M. L. Jambhekar A. Lahav G. (2019). The multiple mechanisms that regulate p53 activity and cell fate. Nat. Rev. Mol. Cell Biol. 20 (4), 199–210. 10.1038/s41580-019-0110-x 30824861

[B16] Han J. Lee J. D. Jiang Y. Li Z. Feng L. Ulevitch R. J. (1996). Characterization of the structure and function of a novel MAP kinase kinase (MKK6). J. Biol. Chem. 271 (6), 2886–2891. 10.1074/jbc.271.6.2886 8621675

[B17] Hernaez R. Kramer J. R. Khan A. Phillips J. McCallister K. Chaffin K. (2022). Depression and anxiety are common among patients with cirrhosis. Clin. Gastroenterol. Hepatol. 20 (1), 194–203.e1. 10.1016/j.cgh.2020.08.045 32835845 PMC8210475

[B18] Jiang R. Wu J. Rosenblatt M. Dai W. Rodriguez R. X. Sui J. (2023). Elevated C-reactive protein mediates the liver-brain axis: a preliminary study. EBioMedicine 93, 104679. 10.1016/j.ebiom.2023.104679 37356206 PMC10320521

[B19] Jo K. W. Lee D. Cha D. G. Oh E. Choi Y. H. Kim S. (2022). Gossypetin ameliorates 5xFAD spatial learning and memory through enhanced phagocytosis against Aβ. Alzheimers Res. Ther. 14 (1), 158. 10.1186/s13195-022-01096-3 36271414 PMC9585741

[B20] Johnson G. L. Lapadat R. (2002). Mitogen-activated protein kinase pathways mediated by ERK, JNK, and p38 protein kinases. Science 298 (5600), 1911–1912. 10.1126/science.1072682 12471242

[B21] Khan A. Manna K. Bose C. Sinha M. Das D. K. Kesh S. B. (2013). Gossypetin, a naturally occurring hexahydroxy flavone, ameliorates gamma radiation-mediated DNA damage. Int. J. Radiat. Biol. 89 (11), 965–975. 10.3109/09553002.2013.811310 23738882

[B22] Khan A. Manna K. Das D. K. Kesh S. B. Sinha M. Das U. (2015). Gossypetin ameliorates ionizing radiation-induced oxidative stress in mice liver--a molecular approach. Free Radic. Res. 49 (10), 1173–1186. 10.3109/10715762.2015.1053878 25994373

[B23] Khan A. Shal B. Naveed M. Shah F. A. Atiq A. Khan N. U. (2019). Matrine ameliorates anxiety and depression-like behaviour by targeting hyperammonemia-induced neuroinflammation and oxidative stress in CCl4 model of liver injury. Neurotoxicology 72, 38–50. 10.1016/j.neuro.2019.02.002 30738807

[B24] Kinoshita M. Uchida T. Sato A. Nakashima M. Nakashima H. Shono S. (2010). Characterization of two F4/80-positive Kupffer cell subsets by their function and phenotype in mice. J. Hepatol. 53 (5), 903–910. 10.1016/j.jhep.2010.04.037 20739085

[B25] Kisseleva T. Brenner D. (2021). Molecular and cellular mechanisms of liver fibrosis and its regression. Nat. Rev. Gastroenterol. Hepatol. 18 (3), 151–166. 10.1038/s41575-020-00372-7 33128017

[B26] Kisseleva T. Cong M. Paik Y. Scholten D. Jiang C. Benner C. (2012). Myofibroblasts revert to an inactive phenotype during regression of liver fibrosis. Proc. Natl. Acad. Sci. U. S. A. 109 (24), 9448–9453. 10.1073/pnas.1201840109 22566629 PMC3386114

[B27] Kulle A. Thanabalasuriar A. Cohen T. S. Szydlowska M. (2022). Resident macrophages of the lung and liver: the guardians of our tissues. Front. Immunol. 13, 1029085. 10.3389/fimmu.2022.1029085 36532044 PMC9750759

[B28] Labenz C. Huber Y. Michel M. Nagel M. Galle P. R. Kostev K. (2020). Nonalcoholic fatty liver disease increases the risk of anxiety and depression. Hepatol. Commun. 4 (9), 1293–1301. 10.1002/hep4.1541 32923833 PMC7471420

[B29] Lisman J. Buzsáki G. Eichenbaum H. Nadel L. Ranganath C. Redish A. D. (2017). Viewpoints: how the hippocampus contributes to memory, navigation and cognition. Nat. Neurosci. 20 (11), 1434–1447. 10.1038/nn.4661 29073641 PMC5943637

[B30] Masuda A. Nakamura T. Abe M. Iwamoto H. Sakaue T. Tanaka T. (2020). Promotion of liver regeneration and anti‑fibrotic effects of the TGF‑β receptor kinase inhibitor galunisertib in CCl4‑treated mice. Int. J. Mol. Med. 46 (1), 427–438. 10.3892/ijmm.2020.4594 32377696

[B31] Oh E. Lee J. Cho S. Kim S. W. Won K. Shin W. S. (2023). Gossypetin prevents the progression of nonalcoholic steatohepatitis by regulating oxidative stress and AMP-activated protein kinase. Mol. Pharmacol. 104 (5), 214–229. 10.1124/molpharm.123.000675 37595967

[B32] Raingeaud J. Whitmarsh A. J. Barrett T. Dérijard B. Davis R. J. (1996). MKK3- and MKK6-regulated gene expression is mediated by the p38 mitogen-activated protein kinase signal transduction pathway. Mol. Cell Biol. 16 (3), 1247–1255. 10.1128/mcb.16.3.1247 8622669 PMC231107

[B33] Rechnagel R. O. Glende E. A. Jr. (1973). Carbon tetrachloride hepatotoxicity: an example of lethal cleavage. CRC Crit. Rev. Toxicol. 2 (3), 263–297. 10.3109/10408447309082019 4357489

[B34] Rodrigo R. Cauli O. Gomez-Pinedo U. Agusti A. Hernandez-Rabaza V. Garcia-Verdugo J. M. (2010). Hyperammonemia induces neuroinflammation that contributes to cognitive impairment in rats with hepatic encephalopathy. Gastroenterology 139 (2), 675–684. 10.1053/j.gastro.2010.03.040 20303348

[B35] Samant N. P. Gupta G. L. (2022). Gossypetin-based therapeutics for cognitive dysfunction in chronic unpredictable stress-exposed mice. Metab. Brain Dis. 37 (5), 1527–1539. 10.1007/s11011-022-00971-0 35377087

[B36] Schwendimann R. N. Minagar A. (2017). Liver disease and neurology. Contin. (Minneap Minn) 23, 762–777. 10.1212/con.0000000000000486 28570328

[B37] Singal A. K. Jampana S. C. Weinman S. A. (2011). Antioxidants as therapeutic agents for liver disease. Liver Int. 31 (10), 1432–1448. 10.1111/j.1478-3231.2011.02604.x 22093324 PMC3228367

[B38] Tanwar S. Rhodes F. Srivastava A. Trembling P. M. Rosenberg W. M. (2020). Inflammation and fibrosis in chronic liver diseases including non-alcoholic fatty liver disease and hepatitis C. World J. Gastroenterol. 26 (2), 109–133. 10.3748/wjg.v26.i2.109 31969775 PMC6962431

[B39] Turning our focus to liver fibrosis (2023). Turning our focus to liver fibrosis. Nat. Rev. Gastroenterol. Hepatol. 20 (10), 625. 10.1038/s41575-023-00844-6 37752274

[B40] Wang C. Ma C. Gong L. Guo Y. Fu K. Zhang Y. (2021). Macrophage polarization and its role in liver disease. Front. Immunol. 12, 803037. 10.3389/fimmu.2021.803037 34970275 PMC8712501

[B41] Wang Z. Du K. Jin N. Tang B. Zhang W. (2023). Macrophage in liver fibrosis: identities and mechanisms. Int. Immunopharmacol. 120, 110357. 10.1016/j.intimp.2023.110357 37224653

[B42] Wen Y. Lambrecht J. Ju C. Tacke F. (2021). Hepatic macrophages in liver homeostasis and diseases-diversity, plasticity and therapeutic opportunities. Cell Mol. Immunol. 18 (1), 45–56. 10.1038/s41423-020-00558-8 33041338 PMC7852525

[B43] Wu G. S. (2004). The functional interactions between the p53 and MAPK signaling pathways. Cancer Biol. Ther. 3 (2), 156–161. 10.4161/cbt.3.2.614 14764989

[B44] Xia S. Huang Y. Zhang Y. Zhang M. Zhao K. Han P. (2023). Role of macrophage-to-myofibroblast transition in chronic liver injury and liver fibrosis. Eur. J. Med. Res. 28 (1), 502. 10.1186/s40001-023-01488-7 37941043 PMC10631085

[B45] Xie X. Liu K. Liu F. Chen H. Wang X. Zu X. (2019). Gossypetin is a novel MKK3 and MKK6 inhibitor that suppresses esophageal cancer growth *in vitro* and *in vivo* . Cancer Lett. 442, 126–136. 10.1016/j.canlet.2018.10.016 30391783

[B46] Xu J. L. Gu J. P. Wang L. Y. Zhu Q. R. You N. N. Li J. (2023). Aberrant spontaneous brain activity and its association with cognitive function in non-obese nonalcoholic fatty liver disease: a resting-state fMRI study. J. Integr. Neurosci. 22 (1), 8. 10.31083/j.jin2201008 36722230

[B47] Yavas E. Gonzalez S. Fanselow M. S. (2019). Interactions between the hippocampus, prefrontal cortex, and amygdala support complex learning and memory. F1000Res 8, F1000 Faculty Rev-1292. 10.12688/f1000research.19317.1 PMC6676505 31448084

[B48] Zhang X. Fan L. Wu J. Xu H. Leung W. Y. Fu K. (2019). Macrophage p38α promotes nutritional steatohepatitis through M1 polarization. J. Hepatol. 71 (1), 163–174. 10.1016/j.jhep.2019.03.014 30914267

[B49] Zhu C. Kim K. Wang X. Bartolome A. Salomao M. Dongiovanni P. (2018). Hepatocyte Notch activation induces liver fibrosis in nonalcoholic steatohepatitis. Sci. Transl. Med. 10 (468), eaat0344. 10.1126/scitranslmed.aat0344 30463916 PMC6822168

